# *anti*-9,10-Di(1-naphth­yl)anthracene pyridine disolvate

**DOI:** 10.1107/S1600536809042706

**Published:** 2009-10-23

**Authors:** Cho Hee Lee, Mi Jong Kim, Yeong-Joon Kim, Jong Tae Je, You-Soon Lee, Sung Kwon Kang

**Affiliations:** a Department of Chemistry, Chungnam National University, Daejeon 305-764, Republic of Korea; b SFC Co Ltd, Ochang TechnoVillage 641-5, Gak-ri, Cheongwon, Chungbuk 363-883, Republic of Korea

## Abstract

In the title compound, C_34_H_22_·2C_5_H_5_N, there is a crystallographic inversion center in the middle of the anthracene ring system. The dihedral angle between the mean planes of the anthracene and naphthalene ring systems is 83.96 (4)°. The crystal structure is stabilized by weak inter­molecular C—H⋯N and C—H⋯π inter­actions.

## Related literature

For general background to blue-light-emitting materials, see: Zhang *et al.* (2003 ▶); Raghunath *et al.* (2006 ▶). For synthetic procedures, see: Kwon *et al.* (2002 ▶); Lee *et al.* (2008 ▶).
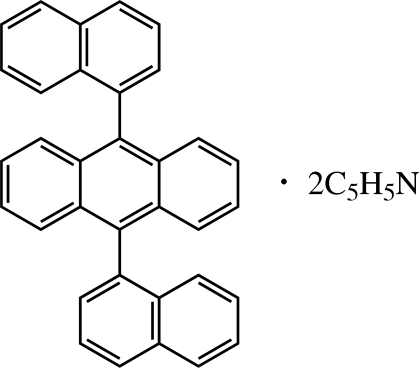


## Experimental

### 

#### Crystal data

C_34_H_22_·2C_5_H_5_N
*M* *_r_* = 588.72
Monoclinic, 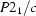

*a* = 8.9810 (18) Å
*b* = 24.166 (5) Å
*c* = 7.2740 (15) Å
β = 93.34 (3)°
*V* = 1576.0 (6) Å^3^
*Z* = 2
Mo *K*α radiation
μ = 0.07 mm^−1^
*T* = 174 K
0.16 × 0.16 × 0.15 mm

#### Data collection

Bruker SMART CCD area-detector diffractometer
Absorption correction: none
16543 measured reflections
3897 independent reflections
3121 reflections with *I* > 2σ(*I*)
*R*_int_ = 0.026

#### Refinement

*R*[*F* ^2^ > 2σ(*F* ^2^)] = 0.044
*wR*(*F* ^2^) = 0.116
*S* = 1.03
3897 reflections
208 parameters
H-atom parameters constrained
Δρ_max_ = 0.27 e Å^−3^
Δρ_min_ = −0.22 e Å^−3^

### 

Data collection: *SMART* (Bruker, 2002 ▶); cell refinement: *SAINT* (Bruker, 2002 ▶); data reduction: *SAINT*; program(s) used to solve structure: *SHELXS97* (Sheldrick, 2008 ▶); program(s) used to refine structure: *SHELXL97* (Sheldrick, 2008 ▶); molecular graphics: *ORTEP-3 for Windows* (Farrugia, 1997 ▶) and *PLATON* (Spek, 2009 ▶); software used to prepare material for publication: *WinGX* publication routines (Farrugia, 1999 ▶).

## Supplementary Material

Crystal structure: contains datablocks global, I. DOI: 10.1107/S1600536809042706/lh2928sup1.cif

Structure factors: contains datablocks I. DOI: 10.1107/S1600536809042706/lh2928Isup2.hkl

Additional supplementary materials:  crystallographic information; 3D view; checkCIF report

## Figures and Tables

**Table 1:** Hydrogen-bond geometry (Å, °)

*D*—H⋯*A*	*D*—H	H⋯*A*	*D*⋯*A*	*D*—H⋯*A*
C13—H13⋯N18^i^	0.93	2.62	3.4887 (19)	156
C4—H4⋯*Cg*1^ii^	0.93	2.86	3.6026 (15)	138
C16—H16⋯*Cg*2^iii^	0.93	2.85	3.6177 (18)	141

Symmetry codes: (i) 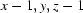; (ii) 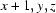; (iii) 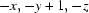. *Cg*1 and *Cg*2 are the centroids of the C9–C14 and C1–C6 rings, respectively.

**Fig. 1.**
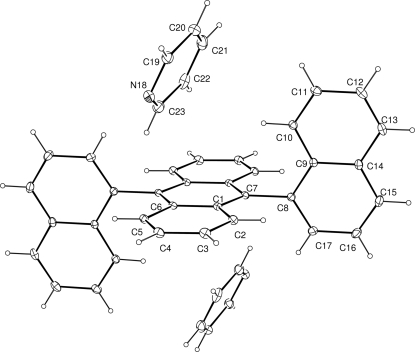
The molecular structure showing the atom-numbering scheme and 30% probability ellipsoids. Unlabeled atoms are related by the symmetry operator (-x, -y+1, -z+1).

**Fig. 2.**
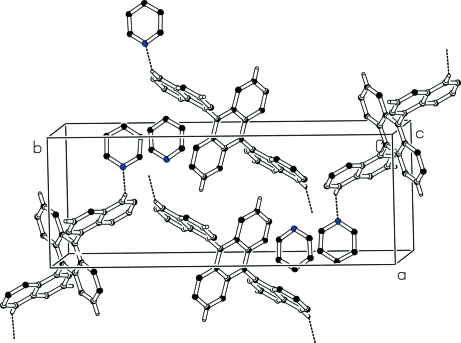
Part of the crystal structure with hydrogen bonds shown as dashed lines. Only H atoms involved in weak C-H···N hydrogen bonds or C-H···π interactions are shown.

## supplementary crystallographic information

### Comment

9,10-Dinaphthylanthracene has been widely used as a blue light emitting material in organic light emitting diodes (OLED) (Zhang *et al.*, 2003; Raghunath *et al.*, 2006). There are two stereoisomers for 9,10-di(1'-naphthyl)anthracene because the single bond rotation about the σ-bond between naphthyl and anthracene moiety is so hindered. Since the two stereoisomers of 9,10-di(1'-naphthyl)anthracene, *syn* and *anti*, could have different physical properties such as electronic states, it is considered important to carry out studies on the isolation and characterization of the isomers. Herein we report the single crystal structure of the anti form of 9,10-di(1'-naphthyl)anthracene. The molecular structure is shown in Fig. 1.

In the title compound, (C_34_H_22_).2(C_5_H_5_N), the dihedral angle between anthracene and naphtyl mean planes is 83.96 (4)°. There is a crystallographic inversion center located in the middle of anthracene ring. The crystal structure is stabilized by weak intermolecular C—H···N and C-H···π interactions (Table 1, Fig. 2).

### Experimental

9,10-Di(1'-naphthyl)anthracene was prepared by a literature procedure (Kwon *et al.*, 2002; Lee *et al.*, 2008). Bromonaphthalene and anthraquinone were commercially available from Aldrich and used as received. n-Buthyllithium (18 ml, 1.6 *M* in hexane) was slowly added at 195K to a THF (50 ml) solution of bromonaphthalene (4.9 g, 0.024 mol). Anthraquinone (2 g, 0.0096 mol) was added to the mixture and the solution was stirred for 3 h at room temperature. Aqueous 2 N HCl solution was added and the organic phase was separated. The organic phase was dried and potassium iodide (0.028 mol), sodium hypophosphite monohydrate (0.0576 mol), and acetic acid (100 ml) were added. The mixture was heated for 4 h under reflux. After cooling, the precipitate was collected, washed with plenty of water, and dried (yield 80%). The separation of two isomers, *syn* and *anti*, was successfully performed after several recrystallizations from toluene and xylene. The single crystal of *anti* form was grown in pyridine and hexane solution.

^1^H NMR (400 MHz, CDCl_3_) δ = 8.09 (d, 2H, J = 8.0), 8.03 (d, 2H, J = 8.0), 7.74 (t, 2H, J = 6.8, J = 7.2), 7.66 (d, 2H, J = 7.2), 7.49 (m, 6H), 7.21(m, 8H).

^13^C NMR (100 MHz, CDCl_3_) δ = 136.8, 135.4, 133.7, 133.7, 130.7, 129.3, 128.3, 128.2, 127.1, 126.8, 126.3, 126.0, 125.6, 125.2.

### Refinement

All H atoms were positioned geometrically and refined using a riding model, with C—H = 0.93 Å, and with *U*_iso_(H) = 1.2*U*_eq_(C)

### Crystal data

**Table:**

C_34_H_22_·2C_5_H_5_N	*F*(000) = 620
*M**_r_* = 588.72	*D*_x_ = 1.241 Mg m^−^^3^
Monoclinic, *P*2_1_/*c*	Mo *K*α radiation, λ = 0.71073 Å
Hall symbol: -P 2ybc	Cell parameters from 5150 reflections
*a* = 8.9810 (18) Å	θ = 2.3–28.1°
*b* = 24.166 (5) Å	µ = 0.07 mm^−^^1^
*c* = 7.2740 (15) Å	*T* = 174 K
β = 93.34 (3)°	Block, colourless
*V* = 1576.0 (6) Å^3^	0.16 × 0.16 × 0.15 mm
*Z* = 2	

### Data collection

**Table:**

Bruker SMART CCD area-detector diffractometer	*R*_int_ = 0.026
φ and ω scans	θ_max_ = 28.3°, θ_min_ = 1.7°
16543 measured reflections	*h* = −11→11
3897 independent reflections	*k* = −31→32
3121 reflections with *I* > 2σ(*I*)	*l* = −9→9

### Refinement

**Table:**

Refinement on *F*^2^	0 restraints
Least-squares matrix: full	H-atom parameters constrained
*R*[*F*^2^ > 2σ(*F*^2^)] = 0.044	*w* = 1/[σ^2^(*F*_o_^2^) + (0.0481*P*)^2^ + 0.4153*P*] where *P* = (*F*_o_^2^ + 2*F*_c_^2^)/3
*wR*(*F*^2^) = 0.116	(Δ/σ)_max_ = 0.001
*S* = 1.03	Δρ_max_ = 0.27 e Å^−^^3^
3897 reflections	Δρ_min_ = −0.22 e Å^−^^3^
208 parameters	

### Special details

**Table:**

Geometry. All e.s.d.'s (except the e.s.d. in the dihedral angle between two l.s. planes) are estimated using the full covariance matrix. The cell e.s.d.'s are taken into account individually in the estimation of e.s.d.'s in distances, angles and torsion angles; correlations between e.s.d.'s in cell parameters are only used when they are defined by crystal symmetry. An approximate (isotropic) treatment of cell e.s.d.'s is used for estimating e.s.d.'s involving l.s. planes.

### Fractional atomic coordinates and isotropic or equivalent isotropic displacement parameters (Å^2^)

**Table:**

	*x*	*y*	*z*	*U*_iso_*/*U*_eq_	
C1	0.05625 (11)	0.54185 (4)	0.38731 (15)	0.0273 (2)	
C2	0.11828 (13)	0.58393 (5)	0.27710 (17)	0.0338 (3)	
H2	0.0574	0.6018	0.188	0.041*	
C3	0.26455 (14)	0.59831 (5)	0.29986 (19)	0.0396 (3)	
H3	0.3027	0.6256	0.2256	0.048*	
C4	0.35912 (13)	0.57214 (5)	0.43571 (19)	0.0389 (3)	
H4	0.4591	0.5822	0.4496	0.047*	
C5	0.30516 (12)	0.53240 (5)	0.54615 (17)	0.0324 (3)	
H5	0.3687	0.5158	0.6353	0.039*	
C6	0.15163 (11)	0.51567 (4)	0.52738 (15)	0.0268 (2)	
C7	−0.09396 (11)	0.52557 (4)	0.36048 (15)	0.0268 (2)	
C8	−0.19254 (12)	0.55196 (5)	0.21282 (15)	0.0294 (2)	
C9	−0.26471 (11)	0.60328 (5)	0.24560 (15)	0.0278 (2)	
C10	−0.24646 (12)	0.63180 (5)	0.41558 (17)	0.0326 (3)	
H10	−0.1844	0.6171	0.51	0.039*	
C11	−0.31893 (14)	0.68071 (5)	0.4428 (2)	0.0424 (3)	
H11	−0.3059	0.6989	0.5553	0.051*	
C12	−0.41311 (14)	0.70367 (5)	0.3013 (2)	0.0459 (3)	
H12	−0.4615	0.737	0.3205	0.055*	
C13	−0.43357 (13)	0.67737 (5)	0.1369 (2)	0.0419 (3)	
H13	−0.4966	0.6929	0.0449	0.05*	
C14	−0.36078 (12)	0.62677 (5)	0.10307 (17)	0.0333 (3)	
C15	−0.38159 (15)	0.59853 (6)	−0.06721 (18)	0.0446 (3)	
H15	−0.4441	0.6136	−0.1607	0.054*	
C16	−0.31118 (16)	0.54967 (7)	−0.09547 (18)	0.0491 (4)	
H16	−0.3257	0.5316	−0.2079	0.059*	
C17	−0.21627 (14)	0.52640 (6)	0.04517 (18)	0.0409 (3)	
H17	−0.1687	0.493	0.0239	0.049*	
N18	0.26262 (13)	0.69991 (6)	0.82489 (18)	0.0519 (3)	
C19	0.19358 (17)	0.74566 (6)	0.8706 (2)	0.0503 (3)	
H19	0.25	0.7778	0.8841	0.06*	
C20	0.04512 (18)	0.74902 (8)	0.8994 (2)	0.0598 (4)	
H20	0.0026	0.7826	0.9302	0.072*	
C21	−0.03967 (18)	0.70250 (9)	0.8825 (2)	0.0664 (5)	
H21	−0.1409	0.7036	0.9029	0.08*	
C22	0.0270 (2)	0.65402 (8)	0.8348 (2)	0.0649 (5)	
H22	−0.0279	0.6215	0.8212	0.078*	
C23	0.1781 (2)	0.65463 (7)	0.8073 (2)	0.0588 (4)	
H23	0.2232	0.6217	0.7748	0.071*	

### Atomic displacement parameters (Å^2^)

**Table:**

	*U*^11^	*U*^22^	*U*^33^	*U*^12^	*U*^13^	*U*^23^
C1	0.0250 (5)	0.0259 (5)	0.0313 (5)	0.0001 (4)	0.0047 (4)	−0.0023 (4)
C2	0.0320 (6)	0.0325 (6)	0.0373 (6)	−0.0009 (4)	0.0052 (5)	0.0035 (5)
C3	0.0358 (6)	0.0362 (6)	0.0479 (7)	−0.0078 (5)	0.0111 (5)	0.0043 (5)
C4	0.0252 (5)	0.0408 (7)	0.0512 (7)	−0.0072 (5)	0.0066 (5)	−0.0028 (6)
C5	0.0238 (5)	0.0333 (6)	0.0402 (6)	−0.0009 (4)	0.0015 (4)	−0.0037 (5)
C6	0.0238 (5)	0.0255 (5)	0.0315 (5)	0.0007 (4)	0.0038 (4)	−0.0040 (4)
C7	0.0252 (5)	0.0254 (5)	0.0298 (5)	0.0015 (4)	0.0019 (4)	−0.0029 (4)
C8	0.0255 (5)	0.0329 (6)	0.0298 (5)	−0.0010 (4)	0.0019 (4)	0.0001 (4)
C9	0.0215 (5)	0.0301 (5)	0.0319 (5)	−0.0034 (4)	0.0015 (4)	0.0034 (4)
C10	0.0269 (5)	0.0322 (6)	0.0383 (6)	−0.0001 (4)	−0.0007 (4)	−0.0020 (5)
C11	0.0362 (6)	0.0365 (7)	0.0546 (8)	0.0003 (5)	0.0033 (6)	−0.0098 (6)
C12	0.0325 (6)	0.0305 (6)	0.0748 (10)	0.0043 (5)	0.0053 (6)	0.0014 (6)
C13	0.0273 (6)	0.0391 (7)	0.0590 (8)	0.0016 (5)	−0.0010 (5)	0.0169 (6)
C14	0.0242 (5)	0.0377 (6)	0.0379 (6)	−0.0032 (4)	−0.0001 (4)	0.0094 (5)
C15	0.0379 (7)	0.0612 (9)	0.0338 (6)	−0.0026 (6)	−0.0071 (5)	0.0092 (6)
C16	0.0494 (8)	0.0666 (10)	0.0303 (6)	−0.0016 (7)	−0.0047 (6)	−0.0088 (6)
C17	0.0407 (7)	0.0451 (7)	0.0366 (7)	0.0031 (5)	0.0006 (5)	−0.0087 (5)
N18	0.0425 (6)	0.0580 (8)	0.0549 (7)	0.0085 (5)	−0.0004 (5)	0.0005 (6)
C19	0.0489 (8)	0.0498 (8)	0.0515 (8)	−0.0012 (6)	−0.0031 (6)	−0.0031 (7)
C20	0.0540 (9)	0.0713 (11)	0.0545 (9)	0.0175 (8)	0.0065 (7)	−0.0048 (8)
C21	0.0395 (8)	0.1033 (15)	0.0563 (10)	−0.0012 (9)	0.0011 (7)	0.0171 (10)
C22	0.0730 (11)	0.0685 (11)	0.0503 (9)	−0.0279 (9)	−0.0201 (8)	0.0170 (8)
C23	0.0767 (11)	0.0469 (9)	0.0515 (9)	0.0112 (8)	−0.0073 (8)	−0.0021 (7)

### Geometric parameters (Å, °)

**Table:**

C1—C7	1.4079 (15)	C12—C13	1.357 (2)
C1—C2	1.4281 (16)	C12—H12	0.93
C1—C6	1.4388 (16)	C13—C14	1.4149 (18)
C2—C3	1.3596 (17)	C13—H13	0.93
C2—H2	0.93	C14—C15	1.4170 (19)
C3—C4	1.4143 (19)	C15—C16	1.361 (2)
C3—H3	0.93	C15—H15	0.93
C4—C5	1.3593 (17)	C16—C17	1.4099 (19)
C4—H4	0.93	C16—H16	0.93
C5—C6	1.4358 (15)	C17—H17	0.93
C5—H5	0.93	N18—C19	1.3197 (19)
C6—C7^i^	1.4057 (15)	N18—C23	1.333 (2)
C7—C6^i^	1.4057 (15)	C19—C20	1.364 (2)
C7—C8	1.4944 (16)	C19—H19	0.93
C8—C17	1.3725 (17)	C20—C21	1.359 (3)
C8—C9	1.4260 (15)	C20—H20	0.93
C9—C10	1.4166 (16)	C21—C22	1.370 (3)
C9—C14	1.4273 (16)	C21—H21	0.93
C10—C11	1.3693 (17)	C22—C23	1.383 (3)
C10—H10	0.93	C22—H22	0.93
C11—C12	1.408 (2)	C23—H23	0.93
C11—H11	0.93		
			
C7—C1—C2	121.55 (10)	C13—C12—H12	119.9
C7—C1—C6	120.10 (10)	C11—C12—H12	119.9
C2—C1—C6	118.35 (10)	C12—C13—C14	121.19 (12)
C3—C2—C1	121.30 (11)	C12—C13—H13	119.4
C3—C2—H2	119.4	C14—C13—H13	119.4
C1—C2—H2	119.4	C13—C14—C15	121.98 (12)
C2—C3—C4	120.49 (11)	C13—C14—C9	118.89 (12)
C2—C3—H3	119.8	C15—C14—C9	119.13 (12)
C4—C3—H3	119.8	C16—C15—C14	120.74 (12)
C5—C4—C3	120.57 (11)	C16—C15—H15	119.6
C5—C4—H4	119.7	C14—C15—H15	119.6
C3—C4—H4	119.7	C15—C16—C17	120.21 (12)
C4—C5—C6	121.07 (11)	C15—C16—H16	119.9
C4—C5—H5	119.5	C17—C16—H16	119.9
C6—C5—H5	119.5	C8—C17—C16	121.42 (13)
C7^i^—C6—C5	121.91 (10)	C8—C17—H17	119.3
C7^i^—C6—C1	119.89 (9)	C16—C17—H17	119.3
C5—C6—C1	118.19 (10)	C19—N18—C23	116.01 (14)
C6^i^—C7—C1	120.01 (10)	N18—C19—C20	124.50 (15)
C6^i^—C7—C8	119.79 (9)	N18—C19—H19	117.8
C1—C7—C8	120.20 (10)	C20—C19—H19	117.8
C17—C8—C9	119.43 (11)	C21—C20—C19	118.96 (16)
C17—C8—C7	120.09 (11)	C21—C20—H20	120.5
C9—C8—C7	120.47 (10)	C19—C20—H20	120.5
C10—C9—C8	122.58 (10)	C20—C21—C22	118.66 (15)
C10—C9—C14	118.34 (11)	C20—C21—H21	120.7
C8—C9—C14	119.07 (10)	C22—C21—H21	120.7
C11—C10—C9	120.96 (11)	C21—C22—C23	118.33 (16)
C11—C10—H10	119.5	C21—C22—H22	120.8
C9—C10—H10	119.5	C23—C22—H22	120.8
C10—C11—C12	120.33 (13)	N18—C23—C22	123.54 (16)
C10—C11—H11	119.8	N18—C23—H23	118.2
C12—C11—H11	119.8	C22—C23—H23	118.2
C13—C12—C11	120.29 (12)		

Symmetry codes: (i) −*x*, −*y*+1, −*z*+1.

### Hydrogen-bond geometry (Å, °)

**Table:**

*D*—H···*A*	*D*—H	H···*A*	*D*···*A*	*D*—H···*A*
C13—H13···N18^ii^	0.93	2.62	3.4887 (19)	156
C4—H4···Cg1^iii^	0.93	2.86	3.6026 (15)	138
C16—H16···Cg2^iv^	0.93	2.85	3.6177 (18)	141

Symmetry codes: (ii) *x*−1, *y*, *z*−1; (iii) *x*+1, *y*, *z*; (iv) −*x*, −*y*+1, −*z*.
